# TrkA is a binding partner of NPM‐ALK that promotes the survival of ALK ^+^ T‐cell lymphoma

**DOI:** 10.1002/1878-0261.12088

**Published:** 2017-06-18

**Authors:** Wenyu Shi, Suraj Konnath George, Bhawana George, Choladda V. Curry, Albina Murzabdillaeva, Serhan Alkan, Hesham M. Amin

**Affiliations:** ^1^ Department of Hematopathology The University of Texas MD Anderson Cancer Center Houston TX USA; ^2^ Department of Hematology Affiliated Hospital of the University of Nantong Jiangsu China; ^3^ Department of Pathology and Immunology Baylor College of Medicine & Texas Children's Hospital Houston TX USA; ^4^ Department of Pathology and Laboratory Medicine Cedars‐Sinai Medical Center Los Angeles CA USA; ^5^ MD Anderson Cancer Center UTHealth Graduate School of Biomedical Sciences Houston TX USA

**Keywords:** CHOP, NGF, NPM‐ALK, T‐cell lymphoma, TrkA

## Abstract

Nucleophosmin‐anaplastic lymphoma kinase‐expressing (NPM‐ALK ^+^) T‐cell lymphoma is an aggressive neoplasm that is more commonly seen in children and young adults. The pathogenesis of NPM‐ALK ^+^ T‐cell lymphoma is not completely understood. Wild‐type ALK is a receptor tyrosine kinase that is physiologically expressed in neural tissues during early stages of human development, which suggests that ALK may interact with neurotrophic factors. The aberrant expression of NPM‐ALK results from a translocation between the *ALK* gene on chromosome 2p23 and the *NPM* gene on chromosome 5q35. The nerve growth factor (NGF) is the first neurotrophic factor attributed to non‐neural functions including cancer cell survival, proliferation, and metastasis. These functions are primarily mediated through the tropomyosin receptor kinase A (TrkA). The expression and role of NGF/TrkA in NPM‐ALK ^+^ T‐cell lymphoma are not known. In this study, we tested the hypothesis that TrkA signaling is upregulated and sustains the survival of this lymphoma. Our data illustrate that TrkA and NGF are expressed in five NPM‐ALK ^+^ T‐cell lymphoma cell lines and TrkA is expressed in 11 of 13 primary lymphoma tumors from patients. In addition, we found evidence to support that NPM‐ALK and TrkA functionally interact. A selective TrkA inhibitor induced apoptosis and decreased cell viability, proliferation, and colony formation of NPM‐ALK ^+^ T‐cell lymphoma cell lines. These effects were associated with downregulation of cell survival regulatory proteins. Similar results were also observed using specific knockdown of TrkA in NPM‐ALK ^+^ T‐cell lymphoma cells by siRNA. Importantly, the inhibition of TrkA signaling was associated with antitumor effects *in vivo*, because tumor xenografts in mice regressed and the mice exhibited improved survival. In conclusion, TrkA plays an important role in the pathogenesis of NPM‐ALK ^+^ T‐cell lymphoma, and therefore, targeting TrkA signaling may represent a novel approach to eradicate this aggressive neoplasm.

**Abbreviations**
Ab: antibody
ATP: adenosine triphosphate
BCL‐2: B‐cell lymphoma 2
BCL‐X_L_: B‐cell lymphoma‐extra large
BrdU: bromodeoxyuridine
CD30L: CD30 ligand
CHOP: cyclophosphamide, doxorubicin, vincristine, prednisone
DMEM: Dulbecco's modified Eagle's medium
DMSO: dimethyl sulfoxide
ELISA: enzyme‐linked immunosorbent assay
ERK: extracellular signal‐regulated kinase
FBS: fetal bovine serum
FFPE: formalin‐fixed and paraffin‐embedded
FLT3: fms‐like tyrosine kinase 3
HEPES: 4‐(2‐hydroxyethyl)‐1‐piperazineethanesulfonic acid
HRP: horseradish peroxidase
IC_50_: half maximal inhibitory concentration
IGF‐IR: type I insulin‐like growth factor receptor
IHC: immunohistochemistry
IP: immunoprecipitation
IRS‐1: insulin receptor substrate 1
JAK2: Janus kinase 2
MAPK: mitogen‐activated protein kinase
NGF: nerve growth factor
NPM‐ALK: nucleophosmin‐anaplastic lymphoma kinase
PBST: phosphate‐buffered saline with Tween 20
PDGFRαα: platelet‐derived growth factor receptor α
PI3K: phosphoinositide 3‐kinase
PI: propidium iodide
PKC: protein kinase C
PLC‐γ: phospholipase C‐γ
PMSF: phenylmethane sulfonyl fluoride
RET: rearranged during transfection
RPMI: Roswell Park Memorial Institute
SE: standard error of the mean
SHC: src homology 2 domain‐containing transforming protein C1
SHP‐1: src homology 2 domain‐containing phosphatase 1
siRNA: small‐interfering ribonucleic acid
STAT3: signal transducer and activator of transcription 3
TMB: 3,3,5,5‐tetra methyl benzidine
TrkA: tropomyosin receptor kinase A
TrkAi: tropomyosin receptor kinase A inhibitor
TrkB: tropomyosin receptor kinase B
WB: western blotting

## 1 Introduction

Nucleophosmin‐anaplastic lymphoma kinase‐expressing (NPM‐ALK^+^) T‐cell lymphoma is an aggressive non‐Hodgkin's lymphoma that is frequently encountered in children and adolescents (Shiota *et al*., 1995). Although conventional CHOP (cyclophosphamide, doxorubicin, vincristine, prednisone)‐based polychemotherapy is able to achieve a high rate of remission, relapse and resistance occur in more than 40% of the patients, and prognosis of these patients remains invariably poor (Brugieres *et al*., 1998; Le Deley *et al*., 2008). The aberrant expression of NPM‐ALK occurs because of a reciprocal chromosomal translocation that induces the fusion of the *ALK* gene on chromosome 2p23 and the *NPM* gene on chromosome 5q35 (Morris *et al*., 1994). NPM‐ALK possesses potent oncogenic potential through interactions with molecules involved in the regulation of cell survival and growth such as JAK/STAT, PI3K/AKT, IRS‐1, IGF‐IR, PLC‐γ, and SHC (Amin and Lai, 2007; Amin *et al*., 2004; Bai *et al*., 1998, 2000; Rassidakis *et al*., 2005; Shi *et al*., 2009, 2013; Zamo *et al*., 2002). Because the expression of ALK is primarily identified in neural tissues at early stages of human development, there is a strong possibility that ALK contributes to neural tissue development through interactions with neurotrophic factors (Souttou *et al*., 2001).

The nerve growth factor (NGF) was the first neurotrophic factor to be identified and characterized (Levi‐Montalcini, 1987). NGF is essential for survival and differentiation of neuronal progenitor cells in the central and peripheral nervous systems (Li *et al*., 1995). The effects of NGF/tropomyosin receptor kinase A (TrkA) in cancer appear to be related to the cell type. For instance, NGF/TrkA induces differentiation, apoptosis, and growth inhibition in neuroblastoma and medulloblastoma (Chou *et al*., 2000; Lavenius *et al*., 1995; Matsushima and Bogenmann, 1993), and high expression of TrkA is considered a favorable prognostic indicator in neuroblastoma (Nakagawara *et al*., 1993). Whereas TrkAI splice variant was found to induce these effects, the TrkAIII splice variant was shown to possess significant oncogenic effects in neuroblastoma (Farina *et al*., 2015; Tacconelli *et al*., 2004). Moreover, the association of TrkA with its ligand NGF leads to the phosphorylation and activation of cascades involving PI3K/AKT and MAPK, which contributes to the survival of pheochromocytoma (Yao and Cooper, 1995). Apart from neural tumors, NGF induces morphological transformation in NIH‐3T3 cells (Cordon‐Cardo *et al*., 1991; Ip *et al*., 1993). Furthermore, deregulation of NGF/TrkA signaling has been shown to be involved in the pathogenesis and survival of non‐neural malignancies including prostate, pancreas, skin, breast, and liver cancers (Lagadec *et al*., 2009; Miknyoczki *et al*., 2002; Oelmann *et al*., 1995; Rasi *et al*., 2007; Shonukan *et al*., 2003). Notably, fewer studies have explored the role of NGF/TrkA signaling in hematological malignancies including NPM‐ALK^+^ T‐cell lymphoma (Bellanger *et al*., 2011; Kim *et al*., 2013; Rao *et al*., 2010).

In this study, we hypothesized that TrkA plays important roles in the pathogenesis of NPM‐ALK^+^ T‐cell lymphoma. To test this hypothesis, we used systematic *in vitro* and *in vivo* experimental approaches. Our results show that TrkA sustains the survival of NPM‐ALK^+^ T‐cell lymphoma through association and interactions with NPM‐ALK, and suggest that inhibition of TrkA signaling could be utilized as a potential strategy to combat this aggressive neoplasm.

## 2 Materials and methods

### 2.1 Cell lines and reagents

Five previously characterized NPM‐ALK^+^ T‐cell lymphoma cell lines: Karpas 299, SR‐786, SU‐DHL‐1, SUP‐M2, and DEL (DSMZ, Braunschweig, Germany), were used in the study (Drexler, 2010). The breast cancer cell line MCF7 and neuroblastoma cell line SK‐N‐AS (ATCC, Manassas, VA, USA) were used as positive controls for the expression of NGF and TrkA, respectively. Human peripheral blood CD3^+^ pan‐T lymphocytes were purchased from StemCell Technologies (catalog number: 70024; Vancouver, BC, Canada). The ALK inhibitor ASP3026 (CT‐ASP302; ChemieTek, Indianapolis, IN, USA) was dissolved in DMSO mixed with H_2_O and HCl (1 : 1).

Cells were maintained in RPMI‐1640 medium (NPM‐ALK^+^ lymphoma cell lines) or Dulbecco's modified Eagle's medium (MCF7, SK‐N‐AS) (Thermo Fisher Scientific, Waltham, MA, USA), supplemented with 10% heat‐inactivated fetal bovine serum (FBS), penicillin (100 U·mL^−1^), streptomycin (100 μg·mL^−1^), and l‐glutamine (2 mm) (all from Sigma Aldrich, St. Louis, MO, USA). Cell cultures were maintained at 37 °C in humidified air with 5% CO_2_. In some experiments, cells were cultured overnight in 0.5–1% FBS. Then, 250 or 500 ng·mL^−1^ recombinant human β‐NGF (256‐GF‐100/CF; R&D Systems, Minneapolis, MN, USA) was added with or without 5 μg·mL^−1^ anti‐TrkA‐neutralizing antibody (AF175; R&D Systems).

### 2.2 Small‐molecule TrkA inhibitor (TrkAi)

Selective targeting of TrkA was achieved using TrkAi [(Z)‐4‐(((2‐oxoindolin‐3‐ylidene)methyl)amino)benzenesulfonamide; C_15_H_13_N_3_O_3_S] (648450; Calbiochem, San Diego, CA, USA) dissolved in DMSO.

### 2.3 Antibodies

The following antibodies were purchased: pTrkA (Tyr^490^; 9141), pALK (Tyr^664^; 3341), pSTAT3 (Tyr^705^; 9131), pIGF‐IR (Tyr^1131^; 3021), IGF‐IR (9750), AKT (9272), and pAKT (Ser^473^; 4051) (Cell Signaling Technology, Danvers, MA, USA); ALK (M7195; Dako, Carpinteria, CA, USA); ALK (ab17127; Abcam, Cambridge, MA, USA); TrkA (sc‐118), ERK1/2 (sc‐94), caspase‐3 (sc‐7272), BCL‐2 (sc‐7382), and BCL‐X_L_ (sc‐8392) (Santa Cruz Biotechnology, Santa Cruz, CA, USA); STAT3 (569388; Calbiochem); TrkA (06‐574) and NGF (04‐1119) (EMD Millipore, Billerica, MA, USA); and β‐actin (A‐2228; Sigma).

### 2.4 Immunoprecipitation (IP) and western blotting (WB)

Cell lysates were collected using standard techniques and lysis buffer that was composed of 25 mm HEPES (pH 7.7), 1.5 mm MgCl_2_, 400 mm NaCl, 2 mm ethylenediaminetetraacetic acid, 0.5% Triton X‐100, 3 mm DTT, 0.1 mm phenylmethane sulfonyl fluoride, phosphatase inhibitor (20 mm β‐GP, 1 mm Na_3_VO_4_; Roche, Indianapolis, IN, USA), and protease inhibitor cocktails (10 μg·mL^−1^ leupeptin, 2 μg·mL^−1^ pepstatin, 50 μg·mL^−1^ antipain, 1 × benzamidine, 2 μg·mL^−1^ aprotinin, 20 μg·mL^−1^ chymostatin, Roche). For WB, proteins (50 μg per well) were purified from total cell lysates or from formalin‐fixed and paraffin‐embedded tissue sections from the mice xenografts (Qproteome FFPE Tissue Kit; Qiagen, Valencia, CA, USA). The proteins were then electrophoresed on 6–12% reducing SDS/PAGE. Separated proteins were transferred to nitrocellulose membranes and were then blocked with 5% nonfat dry milk in phosphate‐buffered saline with Tween 20 buffer (0.1% Tween‐20) and incubated at 4 °C overnight with specific primary antibodies. The horseradish peroxidase (HRP)‐conjugated secondary antibodies (GE Healthcare, Piscataway, NJ, USA) were used at 1 : 2000 dilutions. Protein bands were visualized using a chemiluminescence‐based kit (Amersham Life Sciences, Arlington Heights, IL, USA). IP was performed using the Dynabeads Co‐Immunoprecipitation Kit 143.21D (Invitrogen, Waltham, MA, USA). Briefly, 1.5 mg of the Dynabeads M‐270 Epoxy was coupled with 10 μg of antibody overnight. Next day, 1000 μg of protein extract was added to the bead–antibody complex and incubated for 1 h at 4 °C. After washing with IP buffers, beads were eluted in elution buffer and pH was adjusted by adding Tris/HCl 2 m.

### 2.5 ELISA

An ELISA kit (ab99986; Abcam) was used to measure the NGF levels in 100 μL of the cell lines culture supernatant. According to the manufacturer, this kit detects the pro‐ and active forms of NGF. Recombinant NGF was used to create the standard curve and the concentrations were calculated using the BMG Labtech MARS Data Analysis Software (BMG LABTECH GmbH, Ortenberg, Germany). Assays were performed in triplicate.

### 2.6 MTS assay

Cell viability was evaluated by the One Solution Cell Proliferation Assay (MTS) Kit (G3580; Promega, Madison, WI, USA) (George *et al*., 2014; Shi *et al*., 2009). Cells were seeded into 96‐well plates at a concentration of 1 × 10^4^ cells/well and incubated in the presence of vehicle or the indicated concentration of agents at 37 °C for 48 h. The MTS reagent was added and incubated at 37 °C for 4 h. Measurements were taken using an ELISA plate reader at 490 nm.

### 2.7 Cell proliferation

Cell proliferation was evaluated by bromodeoxyuridine (BrdU) assay using ELISA‐based kit (Roche) (George *et al*., 2014; Shi *et al*., 2009). Briefly, cells were seeded into 96‐well plates at a concentration of 2 × 10^5^ cells/well and incubated for 24 h in the presence of 20 μL of BrdU. Plates were then centrifuged, and the anti‐BrdU antibody, peroxidase goat anti‐mouse IgG conjugate (1 : 2000), and 3,3,5,5‐tetra methyl benzidine (TMB) substrate were added. Reaction was stopped, and plates were read in an ELISA reader at 450/595 nm.

### 2.8 Apoptosis

Quantitative analysis of apoptosis was performed by flow cytometry after staining the cells with annexin V‐FITC and propidium iodide (556547; BD Biosciences, San Jose, CA, USA) (George *et al*., 2014; Shi *et al*., 2009). Moreover, morphologic changes consistent with apoptosis were evaluated in cytospin‐prepared slides from the cell lines after staining with Giemsa.

### 2.9 Colony formation

Methylcellulose medium was prepared by mixing 1% methylcellulose stock (Methocult H4230; StemCell Technologies) with RPMI‐1640 (1 : 4) (George *et al*., 2014; Shi *et al*., 2009). Thereafter, 3.5 mL of methylcellulose medium was added to 15‐mL tubes. Harvested cells were added in a 1 : 10 (v/v) ratio. Tubes were tightly capped, and the mix was gently inverted several times. Then, 3.5 mL of the mix was divided into 24‐well plates in triplicate. Plates were placed in a humidified incubator at 37 °C in 5% CO_2_ for approximately 5 days and then p‐iodonitrotetrazolium violet was added for 24 h. Colonies were visualized using the FluorChem 8800 imaging system (Alpha Innotech, San Leandro, CA, USA).

### 2.10 Tyrosine kinase activity assay

TrkA tyrosine kinase activity in NPM‐ALK^+^ T‐cell lymphoma cells was measured using a commercially available kit (MK410; Takara Bio, Otsu, Shiga, Japan) following the manufacturer's protocol. Cells (2 × 10^6^) were immunoprecipitated by using TrkA antibody incubated overnight at 4 °C. Samples were centrifuged, diluted in kinase reacting buffer, and added in duplicate in a 96‐well microtiter plate containing immobilized synthetic peptide substrates. ATP‐2Na was added to start tyrosine phosphorylation. Subsequently, blocking, addition of anti‐phosphotyrosine (PY20)/HRP solution, and washing steps were performed. Finally, HRP substrate TMB was added, reaction was stopped after 15 min, and the absorbance was measured at 450 nm.

### 2.11 Immunohistochemical (IHC) staining

Studies involving human tissues were performed after approval of the Institutional Review Board. Formalin‐fixed and paraffin‐embedded ALK^+^ T‐cell lymphoma human tumors, mice xenograft tumors, and cellblocks prepared from T lymphocytes and the lymphoma cell lines were probed by IHC for the expression of TrkA (1 : 400; AF175; R&D Systems) using standard techniques (George *et al*., 2014; Shi *et al*., 2009). In addition, IHC was used to study the expression of pIGF‐IR (Tyr^1161^, 1 : 75; ab39398; Abcam) and pSTAT3 (Tyr^705^, 1 : 100; 4113; Cell Signaling Technology) in the mice tumors. Photomicrographs were captured using a Nikon Microphot FXA microscope (Nikon Instruments, Melville, NY, USA) and an Olympus DP70 camera (Olympus America, Melville, NY, USA).

### 2.12 Transfection, plasmids, and siRNA

The *NPM‐ALK* in pCDNA3.1(+) expression plasmid was used as previously described to induce exogenous expression of NPM‐ALK (Shi *et al*., 2009). In addition, SMARTpool‐designed siRNA (Dharmacon, Lafayette, CO, USA) was used to knockdown *ALK*, and the siCONTROL nontargeting siRNA (Dharmacon) was used as a negative control. Cells were microporated using Neon™ Transfection System (Invitrogen) according to the manufacturer's instructions. Briefly, 5 × 10^6^ cells for each transfection were washed with PBS, resuspended in 100 μL of resuspension buffer (R‐buffer), and mixed with plasmid DNA (10 μg) or siRNA. The cells’ DNA/siRNA mixture was subjected to three pulses with pulse width of 10 ms at 1500 V. In some experiments, cells were transfected with the SignalSilence TrkA siRNA I (Cell Signaling Technology) using the Amaxa 4D nucleofection system (solution SF, program CA‐150; Lonza, Atlanta, GA, USA). Cells were incubated for 48 h before performing cellular and biochemical assays.

### 2.13 Lymphoma xenograft tumors

Human lymphoma murine xenograft was utilized to test *in vivo* efficacy of the TrkAi. These experiments were performed after approval of our Institutional Animal Care and Use Committee. SCID/beige mice (5‐ to 6‐week‐old females; Taconic, Cambridge City, IN, USA) were injected s.c. into the left flank with 5 × 10^6^ SR‐786 cells. Tumors developed by day 7. Mice (*n* = 5 per group) were randomly selected for the different treatment regimens. In the first group, TrkAi was injected subcutaneously at a dose of 10 mg·kg^−1^ twice a day for 28 days. In the second group, CHOP at half the conventional dose [cyclophosphamide (20 mg·kg^−1^), doxorubicin (1.65 mg·kg^−1^), and vincristine (0.25 mg·kg^−1^) were given intravenously, and prednisone (0.1 mg·kg^−1^) was given by oral gavage] was administered for the first five consecutive days. In the third group, the combination of TrkAi and the CHOP was used at doses and time points described earlier. Although mice were treated only up to 28 days, they were monitored for tumor growth and progression up to 105 days. Tumor volumes were calculated according to the formula: 0.5 × *a* × *b* ^2^, where ‘*a*’ is the length and ‘*b*’ is the width of the tumor. After euthanasia, total body necropsy was performed and tumors were fixed in 10% buffered formalin or snap‐frozen in liquid nitrogen.

### 2.14 Statistical analysis

Statistical differences between the experimental groups were measured using Student's *t*‐test, one‐way ANOVA, or Dunnett's multiple comparison test when appropriate. A *P *< 0.05 was considered statistically significant. graphpad prism software was used to perform the analysis (GraphPad Software Inc., San Diego, CA, USA).

## 3 Results

### 3.1 The expression of TrkA and NGF in NPM‐ALK^+^ T‐cell lymphoma

**Figure 1:**
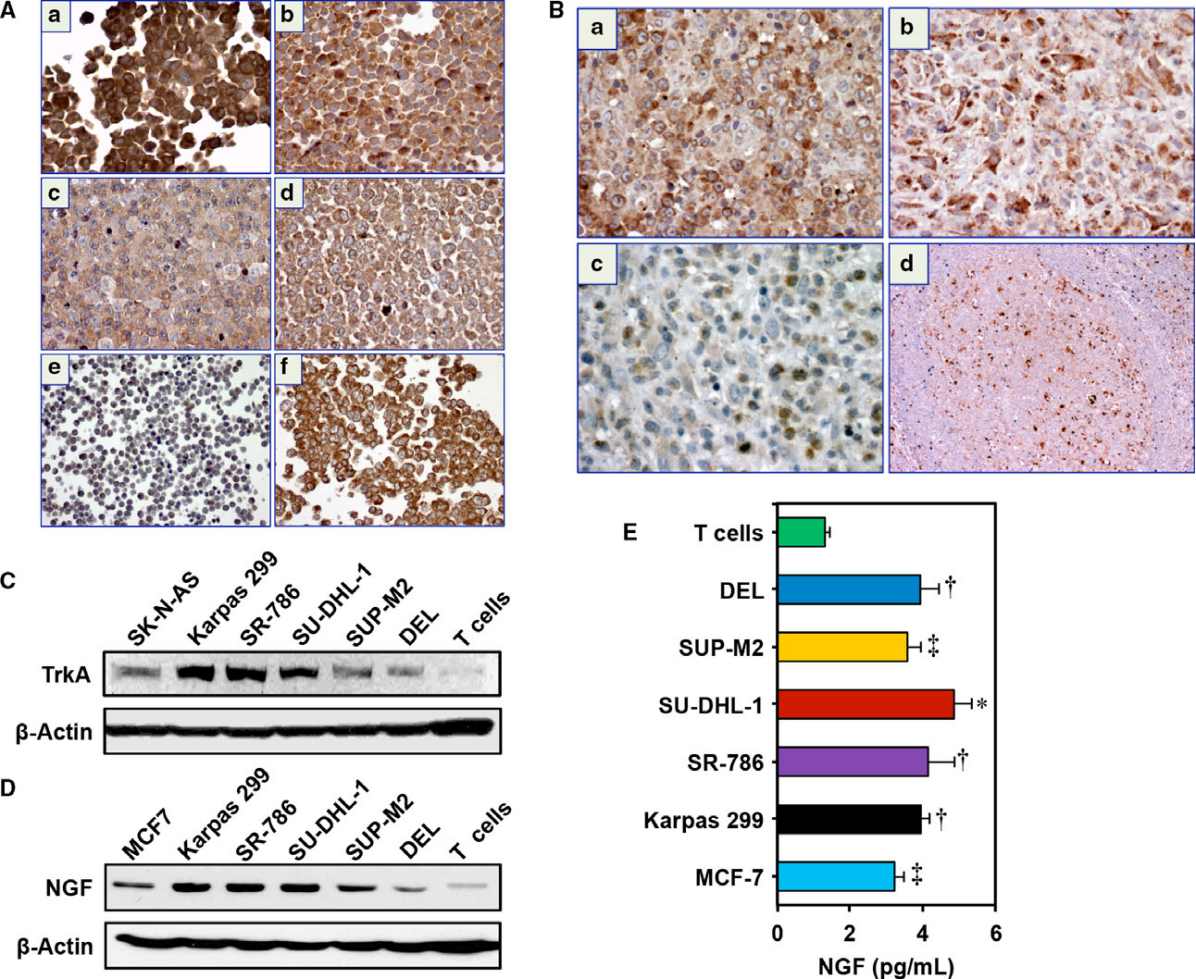
The expression of TrkA and NGF in NPM‐ALK ^+^ T‐cell lymphoma cell lines and human tumors. (A) Immunohistochemical staining shows that TrkA is strongly expressed in the NPM‐ALK ^+^ T‐cell lymphoma cell lines Karpas 299 (a), SU‐DHL‐1 (b), SUP‐M2 (c), and DEL (d). In contrast, only scattered normal human T lymphocytes are weakly positive for TrkA (e). The neuroblastoma cells SK‐N‐AS were used as positive control for the expression of TrkA (f). (B) Two examples of human ALK ^+^ T‐cell lymphoma tumors are shown that are strongly positive for TrkA (a, b). An ALK ^+^ lymphoma tumor that is negative for TrkA (c). Sections from a reactive lymph node demonstrate that TrkA is strongly expressed in dendritic cells and weakly expressed in scattered B lymphocytes residing within the germinal center of a reactive lymphoid follicle. The surrounding mantle zone and interfollicular areas are predominantly negative for TrkA. Original magnification is × 400 in A and B; excluding B (d) where original magnification is × 100. (C) Consistent with IHC data, WB shows that TrkA is highly expressed in five NPM‐ALK ^+^ T‐cell lymphoma cell lines than in normal human T lymphocytes. SK‐N‐AS cells were used as positive control for the expression of TrkA. (D) Compared with human T lymphocytes, the expression of NGF is also increased in NPM‐ALK ^+^ T‐cell lymphoma cell lines, and MCF7 cells were used as positive controls. In C and D, β‐actin shows equal protein loading. MW: TrkA, 140 kDa; NGF, 32 kDa. (E) ELISA demonstrates that the release of NGF from the lymphoma cell lines is significantly higher than from the T cells. MCF7 cells were used as positive control. Data represent means ± SE of three independent experiments (*P < 0.001; ^†^ *P *< 0.01; ^‡^ *P *< 0.05 vs. T cells).

We examined TrkA protein expression in five NPM‐ALK^+^ T‐cell lymphoma cell lines using IHC. Figure 1A shows that TrkA protein was highly expressed in the lymphoma cell lines Karpas 299 (a), SU‐DHL‐1 (b), SUP‐M2 (c), and DEL (d), while normal human T lymphocytes exhibited much weaker expression (e). SK‐N‐AS was used as the positive control for the expression of TrkA (f). We also analyzed the expression of TrkA in 13 primary lymphoma tumors from 11 patients (eight females and three males; age range: 1–16 years; median age: 10 years old). The majority of the primary lymphoma tumors from patients (77%; 11/13 tumors) exhibited significant levels of TrkA; examples are shown in Fig. 1B (a and b). An example of a negative tumor is shown in (c). A reactive lymph node demonstrated the expression of TrkA in dendritic and stromal cells within the germinal center, whereas the mantle zone and interfollicular areas were negative (d).

Using WB, high levels of expression of TrkA were documented in five NPM‐ALK^+^ T‐cell lymphoma cell lines (Fig. 1C). In contrast, T cells exhibited much lower levels of TrkA. SK‐N‐AS cells were used as a positive control for TrkA expression. Likewise, the expression of NGF was upregulated in the lymphoma cell lines, with much lower levels in the T cells (Fig. 1D). The MCF7 cells served as a positive control for the expression of NGF. In order to determine whether NPM‐ALK^+^ T‐cell lymphoma cells secrete detectable levels of NGF, ELISA was performed on cell culture supernatants. Although, according to the manufacturer, the ELISA kit detects both the pro‐ and active forms of NGF, the data support autocrine secretion of NGF by the lymphoma cell lines compared with human T lymphocytes that appear to release much lower levels (Fig. 1E). MCF7 cells were used as a positive control for NGF.

### 3.2 The association and functional interactions between TrkA and NPM‐ALK

**Figure 2:**
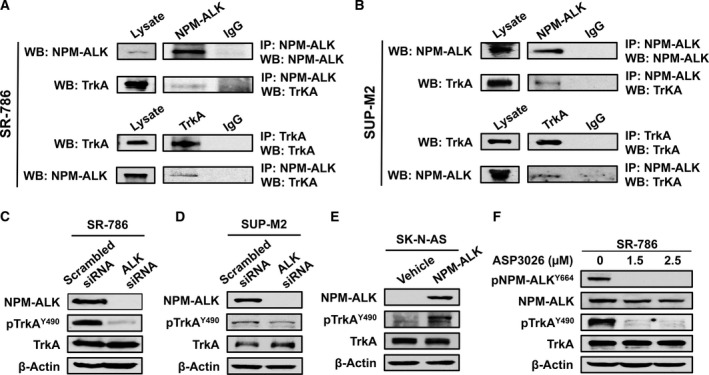
The association and interactions between TrkA and NPM‐ALK. (A) IP using ALK antibody followed by WB using TrkA antibody shows that TrkA and NPM‐ALK are physically associated in SR‐786 (A) and SUP‐M2 cells (B). Similar results were obtained when TrkA and ALK antibodies were used for IP and WB, respectively. Input levels are shown where lysates were probed only with ALK or TrkA antibody. In addition, controls are shown where IgG was used for IP. Similar results were also obtained in Karpas 299 cells (Fig. S1A). (C) and (D) Specific downregulation of NPM‐ALK using ALK siRNA was associated with remarkable decrease in the phosphorylation levels of TrkA in SR‐786 and SUP‐M2 cells, which suggests functional interactions between the two kinases. (E) In agreement with this idea, forced expression of NPM‐ALK in SK‐N‐AS cells was associated with a pronounced increase in TrkA phosphorylation levels. (F) In addition, selective inhibition of ALK kinase activity by treating the SR‐786 cells with ASP3026 for 48 h caused remarkable downregulation of pNPM‐ALK. The decrease in pNPM‐ALK was also associated with downregulation of pTrkA. ASP3026 did not induce changes in the basal levels of NPM‐ALK and TrkA. In WB experiments, β‐actin supports equal protein loading. MW: TrkA and pTrkA, 140 kDa; NPM‐ALK and pNPM‐ALK, 80 kDa.

Thereafter, we tested whether NPM‐ALK and TrkA are physically associated and functionally interact. IP using anti‐ALK antibody followed by WB using anti‐TrkA antibody illustrated the physical association between NPM‐ALK and TrkA in SR‐786 (Fig. 2A), SUP‐M2 (Fig. 2B), and Karpas 299 (Fig. S1A) cells. These findings were consistent when anti‐TrkA and anti‐ALK antibodies were used for IP and WB, respectively. Then, we analyzed whether NPM‐ALK maintains the phosphorylation of TrkA. Downregulation of NPM‐ALK by ALK siRNA in SR‐786 (Fig. 2C) and SUP‐M2 (Fig. 2D) cells decreased pTrkA levels considerably, suggesting functional interactions between these two kinases. To further support that NPM‐ALK phosphorylates TrkA, we exogenously expressed NPM‐ALK in the human neuroblastoma cells SK‐N‐AS that lack this chimeric protein. Overexpression of NPM‐ALK led to a remarkable increase in TrkA phosphorylation (Fig. 2E). In order to examine whether TrkA is a target of NPM‐ALK tyrosine kinase activity, we used an ALK‐selective inhibitor, ASP3026 (George *et al*., 2014). Treatment of the SR‐786 cells with the ALK inhibitor ASP3026 decreased pNPM‐ALK, which was associated with a pronounced decrease in pTrkA (Fig. 2F).

### 3.3 NGF salvages serum‐deprived NPM‐ALK^+^ T‐cell lymphoma cells and induces the phosphorylation of TrkA in these cells

**Figure 3:**
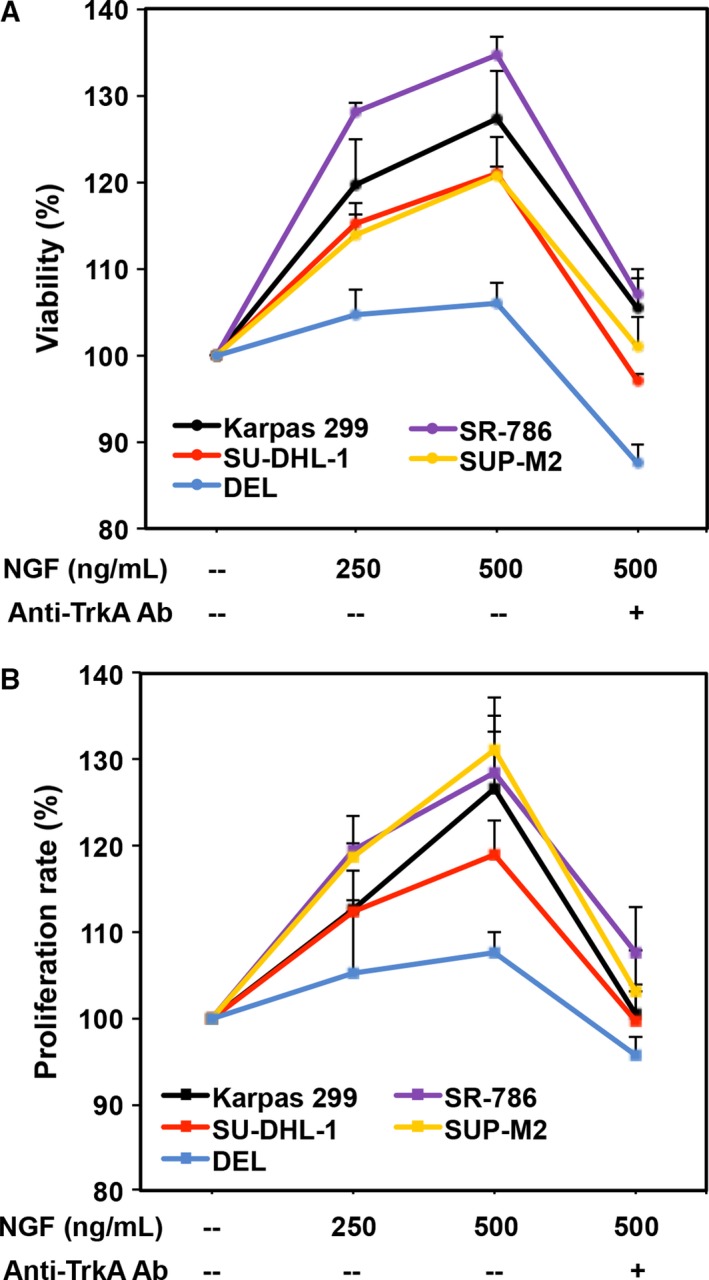
NGF salvages serum‐deprived NPM‐ALK ^+^ T‐cell lymphoma cells. (A) Stimulation of TrkA by NGF increases the viability of serum‐depleted NPM‐ALK ^+^ T‐cell lymphoma cells. At an NGF concentration of 250 ng·mL^−1^, Karpas 299 and SR‐786 cells were more sensitive (*P* < 0.0001 vs. untreated cells) than SU‐DHL‐1 and SUP‐M2 (*P *< 0.01 vs. untreated cells). However, at a concentration of 500 ng·mL^−1^, the increase in cell viability became highly significant in the four cell lines (*P *< 0.0001 vs. untreated cells). Importantly, the increase in cell viability was inhibited in the presence of the anti‐TrkA‐neutralizing antibody (Ab). Although DEL cells showed a similar pattern of response to NGF, the changes did not achieve statistical significance. (B) Similar effects on cell proliferation were detected when the cells were treated with NGF. In this assay, SR‐786 and SUP‐M2 cells were more sensitive as their proliferation increased significantly at a concentration of 250 ng·mL^−1^ (*P *< 0.05). At a concentration of 500 ng·mL^−1^, the increase in cell proliferation became significant in four cell lines (*P* < 0.001 in Karpas 299, SR‐786, SUP‐M2, and *P *< 0.05 in SU‐DHL‐1 vs. untreated cells). The anti‐TrkA‐neutralizing antibody hindered the stimulatory effects of NGF. DEL cells showed a similar pattern of response to NGF but the changes were not statistically significant. The results are shown as means ± SE of three independent experiments. The results are presented as percent change from the baseline (arbitrarily considered as 100%), which represents cells that underwent serum deprivation without the addition of NGF or the anti‐TrkA‐neutralizing antibody.

To further examine the contribution of NGF/TrkA signaling to the survival of NPM‐ALK^+^ T‐cell lymphoma, the lymphoma cell lines were cultured overnight in RPMI supplemented with only 1% FBS. Thereafter, cells were stimulated with NGF for 48 h, in the presence or absence of an anti‐TrkA‐neutralizing antibody. NGF effectively salvaged the serum‐depleted lymphoma cells by increasing their viability (Fig. 3A) and proliferation (Fig. 3B) in a concentration‐dependent manner. Importantly, the effects of NGF were suppressed when cells were simultaneously treated with NGF and an anti‐TrkA‐neutralizing antibody.

Then, we set to examine whether NGF induces the phosphorylation of TrkA in NPM‐ALK^+^ T‐cell lymphoma. Serum‐starved SR‐786 cells were stimulated with NGF (50 ng·mL^−1^) for 24 h in the absence or presence of an anti‐TrkA‐neutralizing antibody. Exogenous NGF slightly increased pTrkA levels, and these effects were inhibited with the addition of the anti‐TrkA‐neutralizing antibody (Fig. S1B). In the absence of exogenous NGF, the addition of the anti‐TrkA‐neutralizing antibody decreased the phosphorylation of TrkA (Fig. S1B), suggesting that it reversed the basal level of phosphorylation of TrkA that most probably resulted from the release of the active form of NGF by SR‐786 cells in the culture medium. Notably, the decrease in pTrkA levels compared with baseline levels was more pronounced than the decrease in pTrkA levels when anti‐TrkA antibody was added to the medium in the presence of exogenous NGF. It is possible that this phenomenon could be due to the smaller amount of NGF secreted by the starved SR‐786 cells vs. when exogenous NGF was added to the culture medium. To further investigate whether NGF secreted by NPM‐ALK^+^ T‐cell lymphoma cells is the active form, conditioned medium from SR‐786 cells was added to serum‐starved SK‐N‐AS cells, which caused modest upregulation of pTrkA in these cells (Fig. S1C). Collectively, these findings suggest that NPM‐ALK^+^ T‐cell lymphoma cells release the active form of NGF.

### 3.4 TrkA signaling is critical for the survival of NPM‐ALK^+^ T‐cell lymphoma

**Figure 4:**
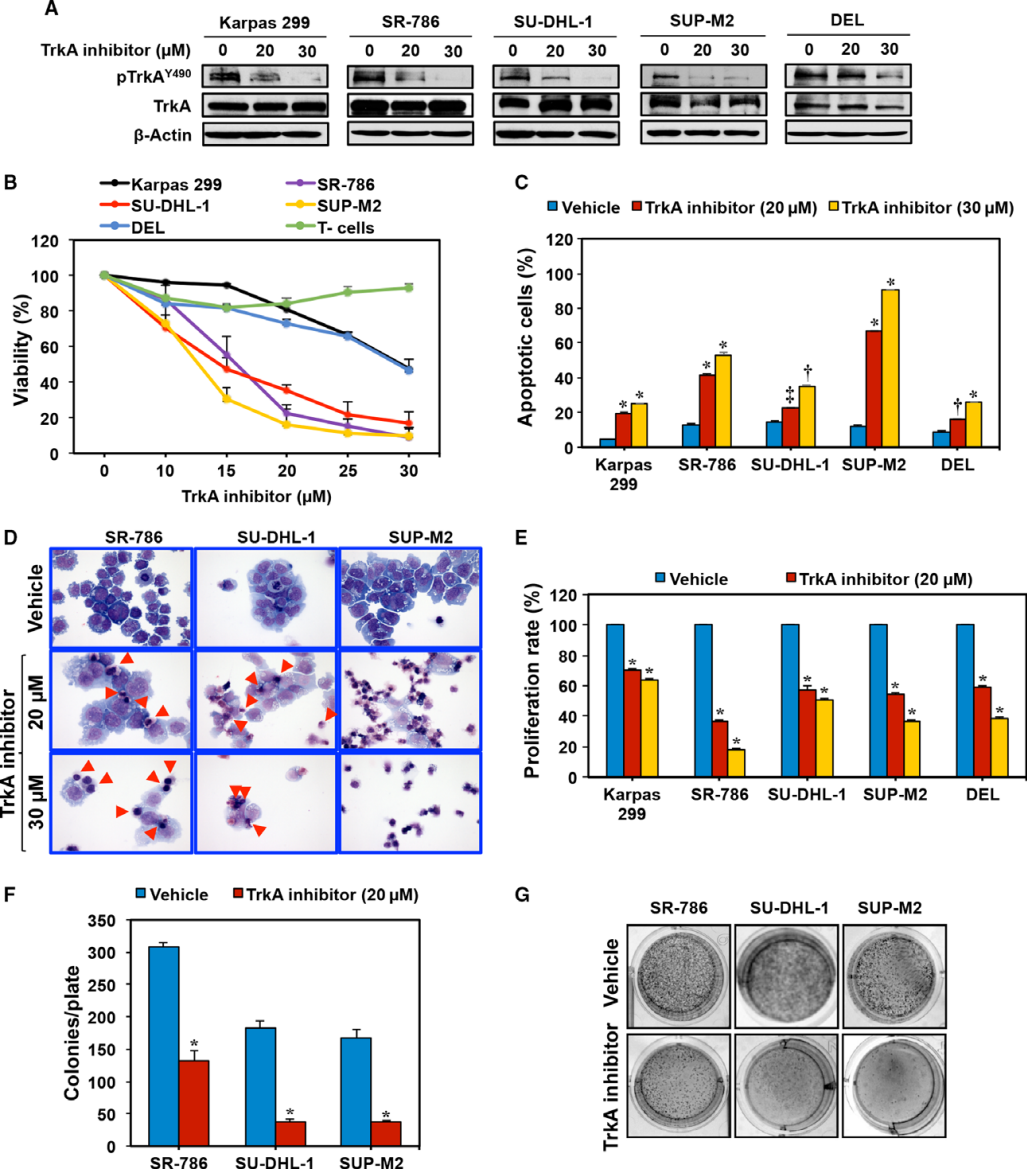
TrkAi decreases pTrkA levels and induces negative cellular effects in NPM‐ALK ^+^ T‐cell lymphoma cells. (A) WB shows that at 48 h TrkAi downregulates the expression of pTrkA in five NPM‐ALK ^+^ T‐cell lymphoma cell lines. Changes are not observed in TrkA or β‐actin. MW: TrkA and pTrkA, 140 kDa. (B) TrkAi‐induced downregulation of pTrkA was associated with a concentration‐dependent decrease in the lymphoma cell viability after 48 h of treatment. The SR‐786, SU‐DHL‐1, and SUP‐M2 cells were more sensitive to the effects of TrkAi (*P* < 0.0001 at 15 μm concentration and above). Although the viability of Karpas 299 and DEL cells decreased after treatment with TrkAi, these two cells appeared to be less sensitive (*P* < 0.0001 at a concentration of 25 μm and above). Importantly, TrkAi has no effect on the viability of the normal human T lymphocytes. (C) Antagonism of TrkA by the inhibitor induces concentration‐dependent increase in apoptotic cells in the lymphoma cells at 48 h. Data represent means ± SE of three independent experiments (*P < 0.0001; ^†^ *P *< 0.001; ^‡^ *P *< 0.01 vs. vehicle‐treated cells). (D) Selective inhibition of TrkA also induced morphological changes consistent with apoptosis in the form of cell shrinkage and nuclear condensation and fragmentation. Red arrowheads mark the apoptotic cells in SR‐786 and SU‐DHL‐1 cells. Almost all of the SUP‐M2 cells that were treated by TrkAi underwent apoptosis. (E) TrkAi induced a significant decrease in the lymphoma cell proliferation at 48 h. The results are shown as means ± SE of three independent experiments (*P < 0.0001 vs. vehicle‐treated cells). (F) TrkAi significantly decreased the anchorage‐independent colony formation potential of SR‐786, SU‐DHL‐1, and SUP‐M2 cells in methylcellulose (means ± SE of three independent experiments; *P < 0.001 vs. vehicle‐treated cells). (G) Representative examples of colonies from the lymphoma cell lines with and without treatment with TrkAi.

Having characterized the expression of TrkA and its association with NPM‐ALK, we set to examine whether inhibition of TrkA may suppress NPM‐ALK^+^ T‐cell lymphoma. A small‐molecule TrkAi was utilized to selectively antagonize TrkA signaling. Treatment of the lymphoma cells with TrkAi for 48 h caused a concentration‐dependent decrease in the levels of pTrkA (Fig. 4A). Data show that at 48 h, inhibition of TrkA was associated with a concentration‐dependent decrease in lymphoma cell viability (Fig. 4B). The IC_50_ was 13.5 μm in SUP‐M2, 15.0 in SU‐DHL‐1, 16.5 in SR‐786, and 30.0 in Karpas 299 and DEL cells. Although TrkAi decreased the viability of Karpas 299 and DEL cells, its effects were more pronounced in SR‐786, SUP‐M2, and SU‐DHL‐1 cells. Importantly, the effects of TrkAi were not detected in the human T cells attesting to the selectivity of this inhibitor.

Quantitative evaluation of apoptosis by flow cytometry displayed that inhibition of TrkA caused a significant increase in apoptotic cells (Fig. 4C). In this regard, the effects of TrkAi at 20 μm concentration were more pronounced in the SUP‐M2, SR‐786, and Karpas 299 cells than in SU‐DHL‐1 and DEL (Fig. 4C). The effects of the TrkAi were more pronounced in all cell lines at a 30 μm concentration. Giemsa staining showed that TrkAi induced morphologic changes consistent with apoptosis, including cell shrinkage and nuclear condensation and fragmentation (Fig. 4D).

In addition, BrdU assay demonstrated a TrkAi concentration‐dependent decrease in cell proliferation (Fig. 4E). To determine whether TrkA inhibition impedes colony‐forming potential of the NPM‐ALK^+^ T‐cell lymphoma, an anchorage‐independent colony formation assay was performed. Treatment with the TrkAi suppressed the ability of SR‐786, SU‐DHL‐1, and SUP‐M2 cells to form colonies in methylcellulose (Fig. 4F). Representative examples of the colonies from each cell line after seven days from treatment are shown (Fig. 4G).

### 3.5 TrkAi decreases TrkA tyrosine kinase activity and induces alterations of survival regulatory proteins in NPM‐ALK^+^ T‐cell lymphoma

**Figure 5:**
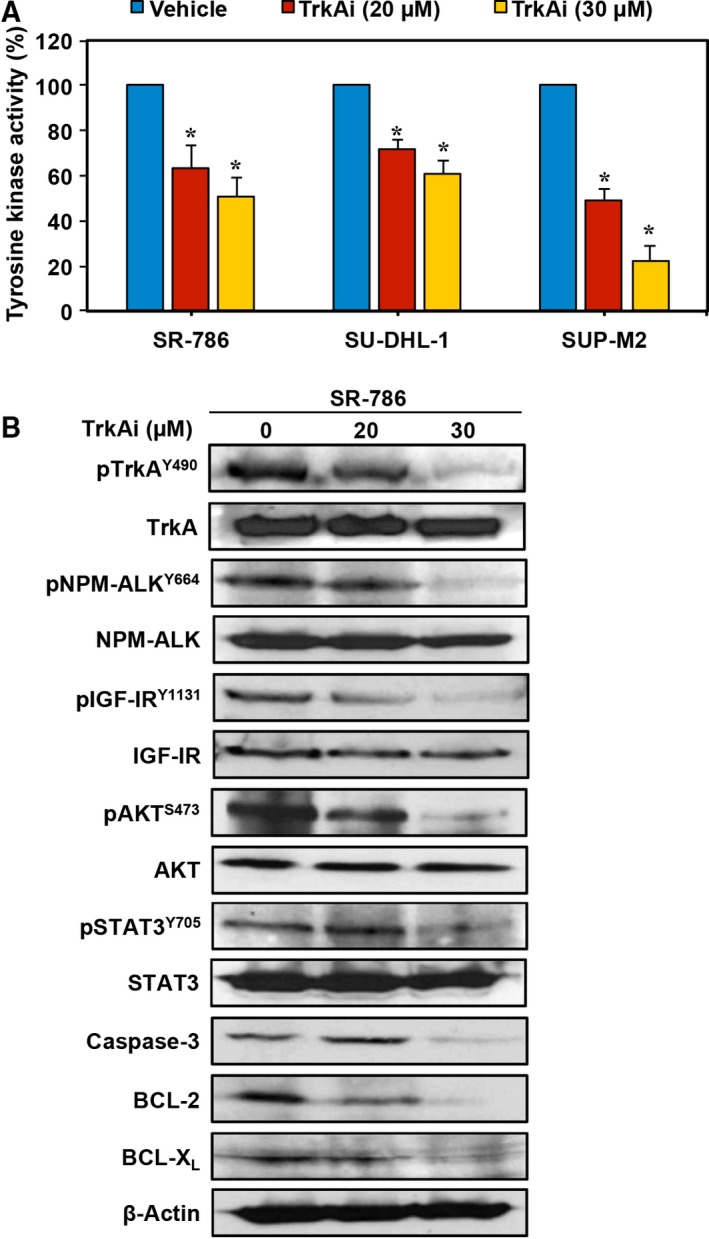
TrkAi efficiently decreases TrkA tyrosine kinase activity and the phosphorylation of survival‐promoting proteins in NPM‐ALK ^+^ T‐cell lymphoma cells. (A) Treatment of SR‐786, SU‐DHL‐1, and SUP‐M2 cells with the TrkAi induced a significant decrease in TrkA tyrosine kinase activity. Results shown are means ± SE of three independent experiments (*P < 0.0001 vs. vehicle‐treated cells). (B) WB shows that at 48 h TrkAi induced downregulation of pTrkA levels in SR‐786 cells. The decrease in pTrkA was associated with a significant decrease in pNPM‐ALK, pIGF‐IR, pAKT, and pSTAT3. These effects were more pronounced at the 30 μm concentration. Changes were not detected in the total protein levels. Moreover, treatment with TrkAi decreased the levels of caspase 3, BCL‐2, and BCL‐X_L_, attesting to apoptosis occurrence. β‐Actin was used as the loading control. MW: TrkA and pTrkA, 140 kDa; NPM‐ALK and pNPM‐ALK, 80 kDa; IGF‐IR and pIGF‐IR, 95 kDa; AKT and pAKT, 60 kDa; STAT3 and pSTAT3, 86 kDa; caspase 3, 32 kDa; BCL‐2, 26 kDa; BCL‐X_L_, 30 kDa.

We first examined the effects of the TrkAi on tyrosine kinase activity. Treatment of SR‐786, SU‐DHL‐1, and SUP‐M2 cells with this inhibitor decreased significantly TrkA tyrosine kinase activity (Fig. 5A). In addition, WB was performed to analyze the changes in survival‐promoting proteins in SR‐786 cells. TrkAi decreased the phosphorylation of TrkA, NPM‐ALK, IGF‐IR, AKT, and STAT3 (Fig. 5B). Consistent with the occurrence of apoptosis, TrkAi caused pronounced decrease in caspase‐3, BCL‐2, and BCL‐X_L_ levels (Fig. 5B).

### 3.6 Specific downregulation of TrkA by siRNA suppresses NPM‐ALK^+^ T‐cell lymphoma survival

**Figure 6:**
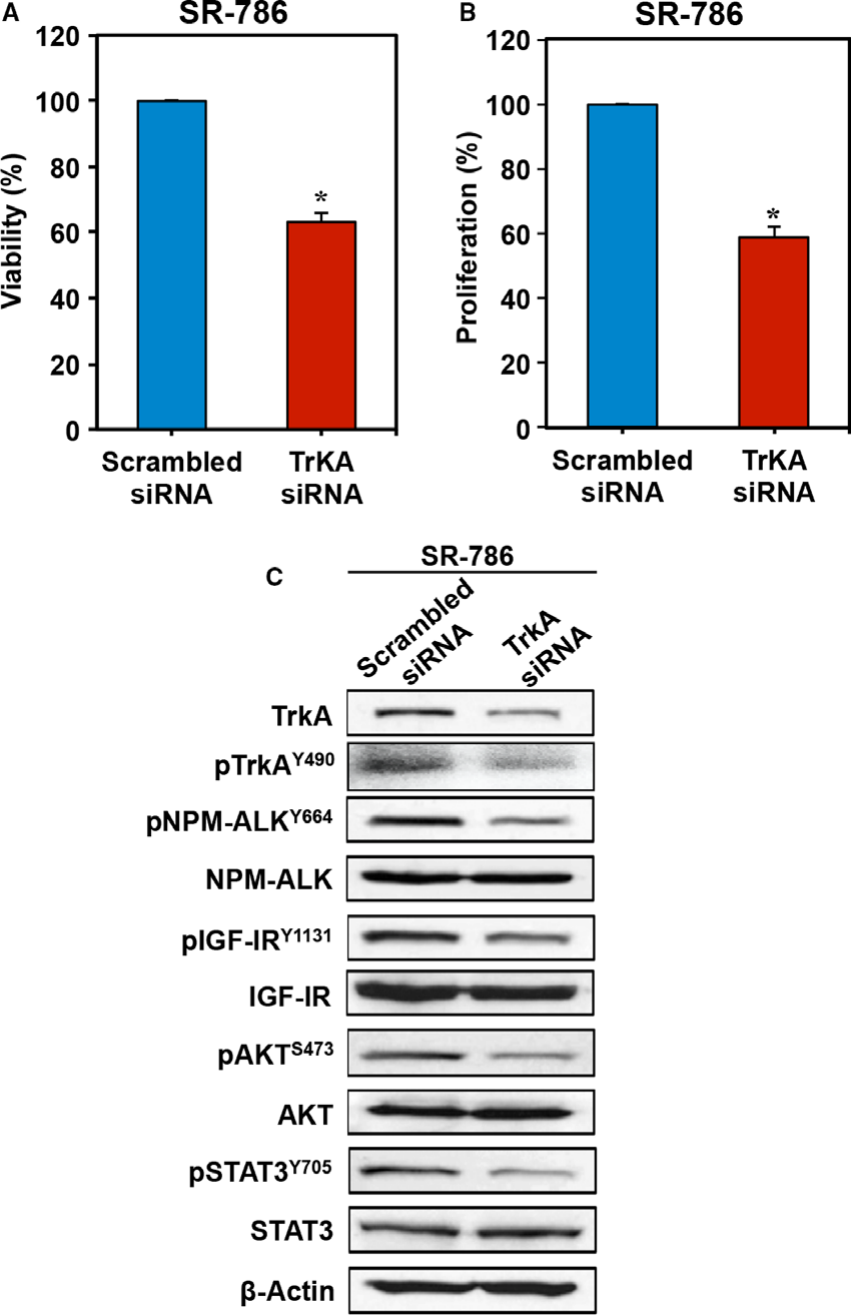
Specific blockade of TrkA by siRNA decreases cell viability and proliferation and downregulates the phosphorylation of survival‐promoting proteins in NPM‐ALK ^+^ T‐cell lymphoma cells. Transfection of TrkA siRNA decreased the (A) viability and (B) proliferation of SR‐786 cells. Data represent means ± SE of three independent experiments (*P < 0.001 vs. scrambled siRNA‐transfected cells). (C) The decrease in TrkA was associated with decreased phosphorylation of NPM‐ALK, IGF‐IR, AKT, and STAT3. β‐Actin was used as the loading control. MW: TrkA and pTrkA, 140 kDa; NPM‐ALK and pNPM‐ALK, 80 kDa; IGF‐IR and pIGF‐IR, 95 kDa; AKT and pAKT, 60 kDa; STAT3 and pSTAT3, 86 kDa.

To further analyze the contribution of TrkA to NPM‐ALK^+^ T‐cell lymphoma cell survival, we induced specific targeting of TrkA by siRNA. Transfection of SR‐786 with TrkA siRNA was associated with a significant decrease in cell viability (Fig. 6A) and proliferation (Fig. 6B). In order to elucidate the signaling mechanisms impacted by the knockdown of TrkA, changes in several survival regulatory proteins were monitored. The siRNA‐induced downregulation of TrkA and pTrkA proteins was associated with decreased phosphorylation of NPM‐ALK, IGF‐IR, AKT, and STAT3 (Fig. 6C).

### 3.7 Targeting TrkA signaling inhibits NPM‐ALK^+^ T‐cell lymphoma xenograft tumor growth in mice

**Figure 7:**
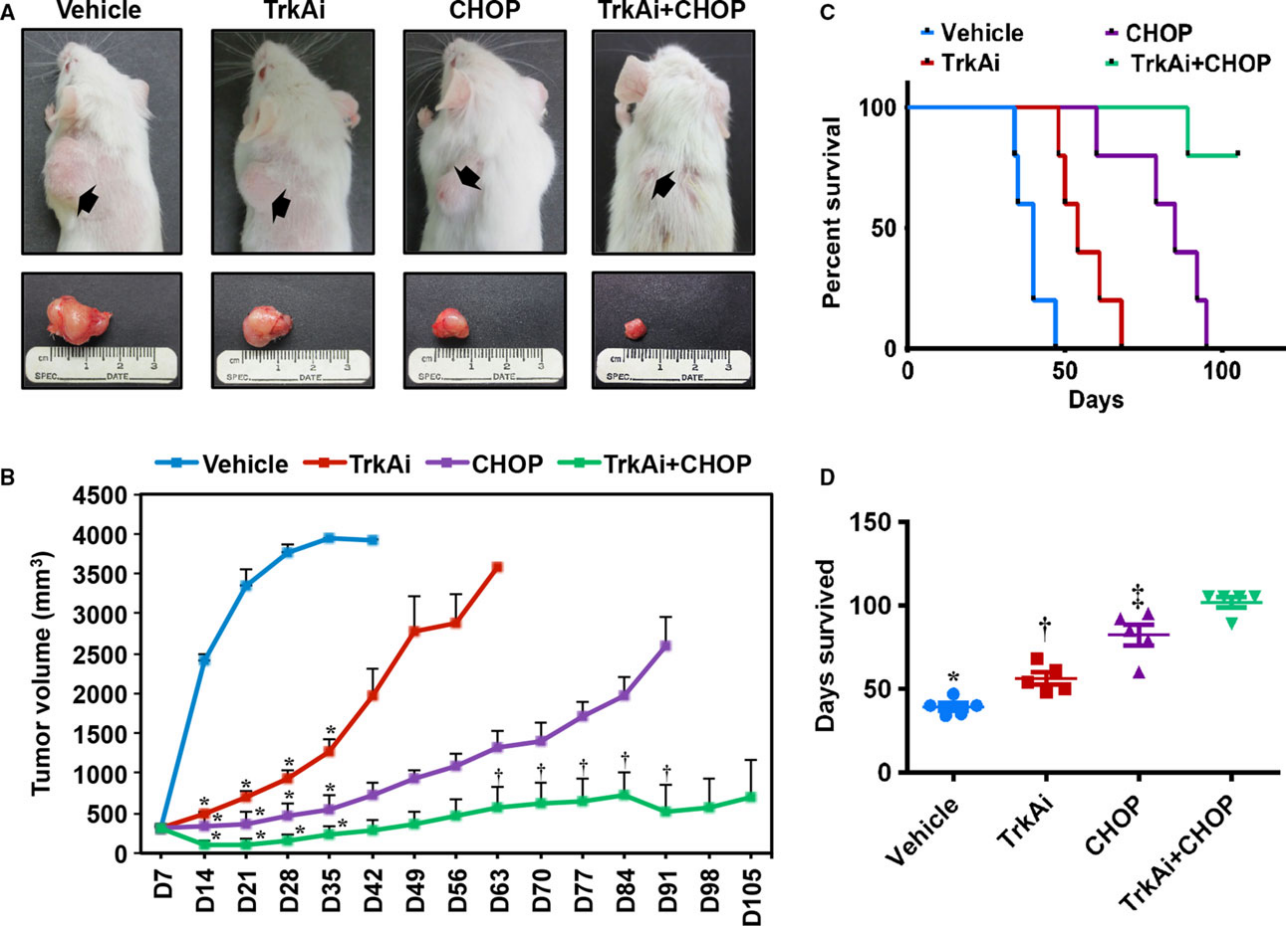
Effects of inhibition of TrkA on NPM‐ALK ^+^ T‐cell lymphoma growth *in vivo*. (A) Tumor sizes of the SR‐786 subcutaneous xenografts treated with vehicle, TrkAi, CHOP, or CHOP+TrkAi. Tumors are depicted before (upper panel) and after (lower panel) dissection. Whereas treatment with TrkAi or CHOP alone decreased tumor sizes, this effect was much more pronounced when mice were simultaneously treated with TrkAi and CHOP. (B) Changes in tumor volume over a period of 105 days in the vehicle‐, TrkAi‐, CHOP‐, CHOP+TrkAi‐treated mice. Data represent means ± SE (*P < 0.0001; ^†^ *P *< 0.05 vs. vehicle‐treated group). (C) Kaplan–Meier survival curve demonstrates significant differences in the survival of the mice treated with vehicle, inhibitor, CHOP, and CHOP+TrkAi. All treatment groups were statistically significant vs. control group in terms of survival (*P *< 0.001). CHOP treatment was slightly superior to using TrkAi alone (*P *< 0.05), but CHOP+TrkAi was far superior to TrkAi or CHOP alone (*P* < 0.005). (D) Scatter plot shows median survival of animals treated with various agents. Data shown represent means ± SE (*P < 0.05 vs. TrkAi, *P *< 0.0001 vs. CHOP and CHOP+TrkAi; ^†^ *P *< 0.05 vs. CHOP,* P *< 0.0001 vs. CHOP+TrkAi; ^‡^ *P *< 0.05 vs. CHOP+TrkAi).

The *in vivo* antitumor activity of TrkAi alone or in combination with CHOP was evaluated in NPM‐ALK^+^ T‐cell lymphoma xenograft tumors. Injections (s.c.) of SR‐786 cells at the mice flanks resulted in tumor nodules by day 7. Representative tumors before and after dissection from the vehicle, TrkAi, CHOP, and TrkAi+CHOP groups at the end of experiments are shown in Fig. 7A. The vehicle group demonstrated the largest tumor size. Whereas TrkAi or CHOP reduced the tumor volume, treating the mice with TrkAi and CHOP resulted in a remarkable decrease in tumor volume. Figure 7B shows the changes in tumor volumes that were assessed every seven days. Vehicle‐treated mice showed a fast, progressive tumor growth, and they were euthanized because of tumor burden. Treatment with TrkAi or CHOP was associated with a significant decrease in the tumor volume particularly at the early stages of the experiment up to day 35. Although tumor growth increased afterward, it was still much less than in the vehicle group. Importantly, combining TrkAi and CHOP suppressed significantly the lymphoma growth throughout the entire duration of the experiment.

Kaplan–Meier curve representing mice survival is shown in Fig. 7C. As indicated, all vehicle‐treated mice were euthanized by day 47, while TrkAi‐ and CHOP‐treated mice survived until days 68 and 95, respectively. In the combination group, four of five mice survived beyond 105 days. The median survival of vehicle‐, TrkAi‐, CHOP‐, and TrkAi+CHOP‐treated mice was 40, 54, 85, and > 105 days, respectively (Fig. 7D).

### 3.8 TrkA inhibition causes downregulation of pNPM‐ALK and downstream effectors *in vivo*

**Figure 8:**
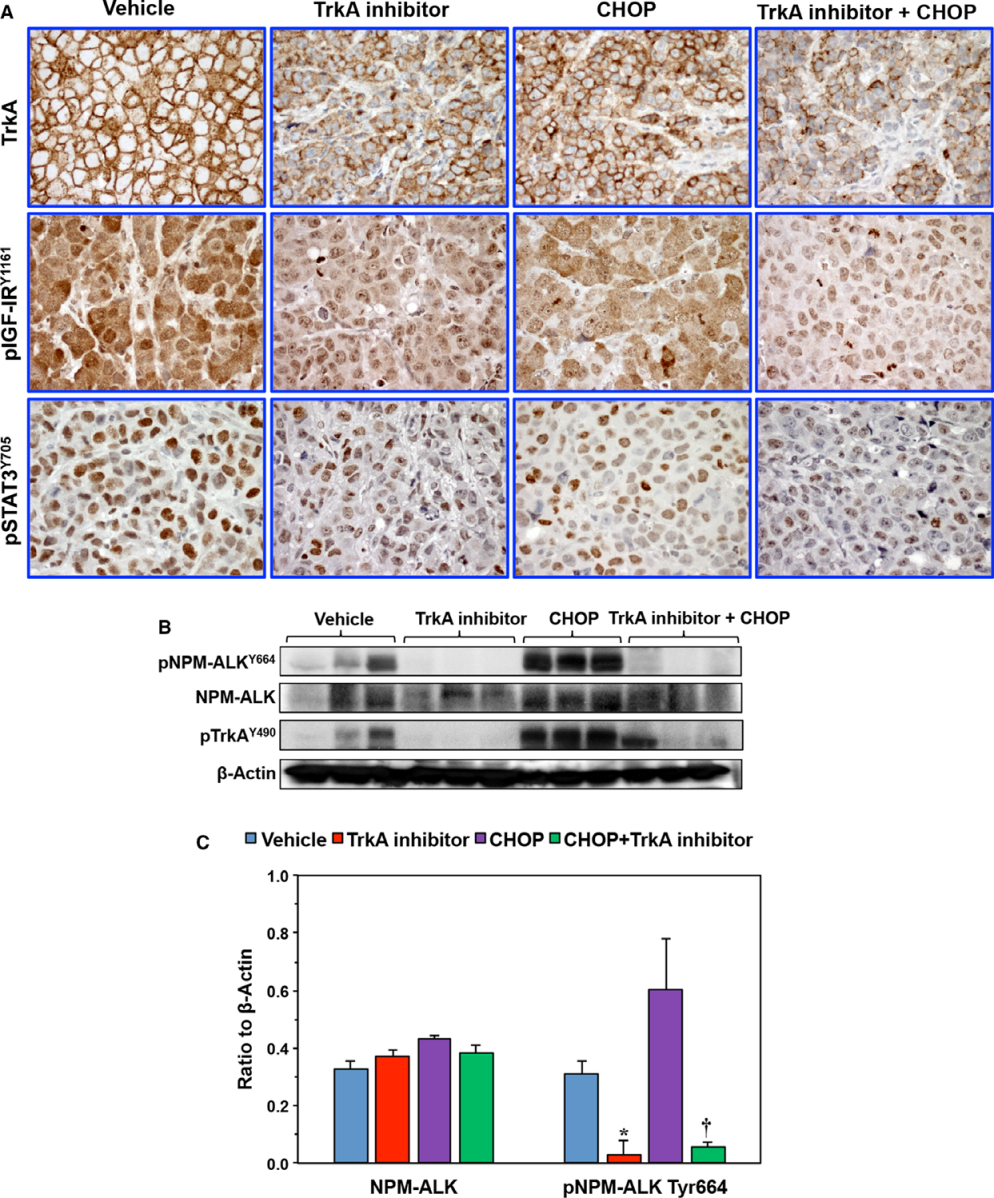
Analysis of lymphoma xenograft tumors. (A) IHC shows high levels of expression of TrkA, pIGF‐IR, and pSTAT3 in the tumors from control mice. Whereas the TrkAi decreased the expression of TrkA, CHOP alone had lesser effects. Combining TrkAi and CHOP induced remarkable downregulation of TrkA levels in the tumors. In addition, the combination regimen remarkably reduced the levels of pIGF‐IR and pSTAT3. Original magnification is × 400. (B) Because of the lack of adequate antibodies to detect pTrkA and pNPM‐ALK expression using IHC, mouse tumors were subjected to protein extraction and WB assay to analyze pNPM‐ALK expression. CHOP alone failed to decrease the levels of pTrkA or pNPM‐ALK in two representative tumors. In contrast, TrkAi alone or in combination with CHOP caused a marked reduction in pTrkA and pNPM‐ALK levels. β‐Actin shows equal protein loading. (C) Densitometry of the NPM‐ALK and pNPM‐ALK bands is shown (*P < 0.05 and ^†^ *P *< 0.01 vs. control). Data shown represent means ± SE of three different xenograft tumors (MW: NPM‐ALK and pNPM‐ALK, 80 kDa; pTrkA, 140 kDa).

A possible mechanism involving the antitumor activity of lymphoma tumor growth was deduced by analyzing the changes in the expression of TrkA and pTrkA in lymphoma tissues from the four groups. In addition, the expression of important survival‐promoting proteins in these tissues, including pNPM‐ALK, pIGF‐IR, and pSTAT3, was also analyzed. IHC and WB showed that TrkAi alone remarkably decreased the expression of pTrkA, pNPM‐ALK, pIGF‐IR, and pSTAT3 (Fig. 8A–C). Whereas treatment with CHOP alone was associated with decreased pIGF‐IR and pSTAT3 levels, it had no effects on pTrkA or pNPM‐ALK. Notably, the effects of combining TrkAi and CHOP on these protein levels were more pronounced.

## 4 Discussion

In this paper, we show for the first time that the expression of TrkA and its ligand NGF is upregulated in the NPM‐ALK^+^ T‐cell lymphoma. In contrast, normal human T lymphocytes demonstrate much lower levels of expression of these proteins. Of important note, our data suggest that NGF/TrkA signaling maintains the survival of NPM‐ALK^+^ T‐cell lymphoma cells. In support of this idea, we found that TrkA is physically associated with NPM‐ALK, which is considered the major oncogenic protein driving the survival of these lymphoma cells. When NPM‐ALK was downregulated by using siRNA or its kinase activity was inhibited by using ASP3026, the phosphorylation of TrkA decreased, suggesting that TrkA may represent a target for NPM‐ALK phosphorylation and tyrosine kinase activity. In addition, forced expression of NPM‐ALK in the neuroblastoma cells SK‐N‐AS that lack this chimeric protein increased significantly the phosphorylation of TrkA.

We also demonstrated autocrine secretion of NGF, the primary ligand of TrkA, in cell culture supernatants of NPM‐ALK^+^ T‐cell lymphoma cell lines, which further proposes a biological role of TrkA signaling. Upon serum deprivation, exogenous NGF was capable of promoting the viability and proliferation of the lymphoma cells. Of note, the effects of NGF were significantly reduced in the presence of an anti‐TrkA antibody, attesting the effects of exogenous NGF were indeed mediated via the TrkA receptor. This trend was previously observed in other types of human cancer cells when NGF significantly enhanced their proliferation and clonal growth (Descamps *et al*., 1998; Oelmann *et al*., 1995; Tsunoda *et al*., 2006). In addition, in our hands, exogenous NGF and conditioned medium from the lymphoma cells increased, albeit slightly, basal levels of phosphorylation of TrkA. These effects were diminished when an anti‐TrkA neutralizing antibody was used simultaneously with NGF. Whereas these results suggest that the lymphoma cells secrete the active form of NGF, phosphorylation of TrkA appears to be more dependent on NPM‐ALK and to a lesser extent on NGF. Selective and specific targeting of TrkA by TrkAi and siRNA, respectively, decreased the viability, proliferation, and anchorage‐independent colony formation, and induced apoptosis in the lymphoma cells. The contribution of TrkA to the survival of NPM‐ALK^+^ T‐cell lymphoma as well as potential utilization of TrkA inhibitors to treat this lymphoma has been further illustrated in the *in vivo* experiments.

The expression and role of ALK as a candidate receptor for regulating critical events during developmental stages of neural tissues have been previously emphasized (Iwahara *et al*., 1997; Souttou *et al*., 2001). These observations imply that ALK may interact with neurotrophic factors to regulate key physiological processes including proliferation, differentiation, and programmed cell death. Moreover, deregulation of ALK has been reported in neural cancers such as neuroblastoma (Chen *et al*., 2008), which signifies that ALK might also have complementary interactions with other neurotrophic factors to regulate processes important for cancer cell survival. In line with this possibility, we found increased expression of TrkA in the NPM‐ALK^+^ T‐cell lymphoma cell lines and in the majority of the human tumors. Functionally, NPM‐ALK appears to induce the phosphorylation of TrkA, which reciprocally maintains the phosphorylation of NPM‐ALK. These findings implicate a proactive role for TrkA/NPM‐ALK axis in promoting the survival of NPM‐ALK^+^ T‐cell lymphoma. Previous studies suggested similar effects of other oncogenic protein kinases in this lymphoma (Amin *et al*., 2003; Cussac *et al*., 2004; Shi *et al*., 2009).

The utility of TrkA as a potential therapeutic target in NPM‐ALK^+^ T‐cell lymphoma was underscored when blockade of TrkA signaling using a small‐molecule inhibitor decreased cell proliferation, viability, and anchor‐independent colony formation of these lymphoma cells. In addition, TrkAi caused these cells to undergo apoptotic cell death. These results support the notion that TrkA signaling might play important roles in maintaining the survival of NPM‐ALK^+^ T‐cell lymphoma. The TrkAi was efficient in reducing the tyrosine phosphorylation and tyrosine kinase activity of TrkA. Our results also show that downregulation of TrkA by TrkAi was associated with decreased phosphorylation of NPM‐ALK, IGF‐IR, STAT3, and AKT in a concentration‐dependent manner. In addition, targeting TrkA induced a significant decrease in caspase‐3, BCL‐2, and BCL‐X_L_, which was consistent with the occurrence of apoptosis. Notably, some variability in the cellular response to TrkAi was observed among the different lymphoma cell lines. This variability could be explained, at least in part, by the intrinsic biochemical differences among these cell lines (Turturro *et al*., 2001). Importantly, the cellular and biochemical effects of selective inhibition of TrkA by TrkAi were consistent when specific downregulation of TrkA was achieved by using siRNA.

It has been shown that TrkA small‐molecule inhibitors, such as GTx‐186, HS‐345, CEP‐751, and CEP‐701, decrease the proliferation and growth of pancreatic, ovarian, prostate, thyroid, neuroblastoma, and medulloblastoma cancer cells (Camoratto *et al*., 1997; Dionne *et al*., 1998; Evans *et al*., 1999; Narayanan *et al*., 2013; Seo *et al*., 2013). GTx‐186 is of particular interest because in a previous report this compound decreased significantly NPM‐ALK tyrosine phosphorylation in Karpas 299 and SU‐DHL‐1 cells (Narayanan *et al*., 2013), similar to the TrkAi‐induced downregulation of NPM‐ALK phosphorylation observed in the current study. Further investigations were not performed to delineate the previously reported results (Narayanan *et al*., 2013). Although it cannot be ruled out that TrkAi induces nonselective effects on NPM‐ALK, the significant decrease in NPM‐ALK phosphorylation after specific downregulation of TrkA by siRNA strongly suggests that NPM‐ALK and TrkA interact reciprocally to sustain their phosphorylation/activation status, and the decrease in pNPM‐ALK was secondary to the decrease in pTrkA.

Previous studies demonstrated that the TrkA inhibitor CEP‐701 (lestaurtinib) and its parent compound CEP‐751 are not highly selective toward TrkA. Indeed, these compounds induce significant inhibitory effects on TrkB and non‐Trk targets including JAK2, FLT3, RET, PKC, and PDGFRα (Brown *et al*., 2006; Hexner *et al*., 2008; Santos *et al*., 2010). Of important consideration to our studies, CEP‐701 also increases CD30L, which could support the survival of NPM‐ALK^+^ T‐cell lymphoma (Atsaves *et al*., 2014). Although the CEP‐751‐related compounds have been utilized in clinical trials of patients with cancer (Chan *et al*., 2008; Hexner *et al*., 2014; Knapper *et al*., 2006; Minturn *et al*., 2011; Santos *et al*., 2010), we decided not to use these compounds in our study because of the concerning issues listed above. Instead, we used the TrkAi, which has been recently shown to induce significant inhibitory effects on TrkA without known effects on other targets (Kokona *et al*., 2012). This TrkAi induces its effects in normal retina cells from rats at a concentration of 10.0 μm, and when a concentration of 1.0 μm was used, only partial effects were observed (Kokona *et al*., 2012). In addition, a recent paper showed that TrkAi induced its cellular effects in the pheochromocytoma cells PC12 at a concentration of 15 μm (Colombo *et al*., 2014). Thus, in the current study, we used this TrkAi at a concentration range of 10–30 μm. Of important note, at this concentration range, TrkAi did not decrease the viability of human T lymphocytes, which implicates a high selectivity of this inhibitor.

In our *in vivo* experiments, TrkAi alone significantly diminished NPM‐ALK^+^ T‐cell lymphoma xenograft tumor size, consistent with the *in vitro* effects of TrkAi and TrkA siRNA. TrkAi also improved the survival of mice with lymphoma tumors. A current frontline therapeutic protocol for NPM‐ALK^+^ T‐cell lymphoma is the CHOP polychemotherapy. Alas, severe adverse reactions, including therapy‐related death, are sometimes encountered during clinical utilization of CHOP (Fisher *et al*., 1993; Lim *et al*., 2010; Sitzia *et al*., 1997; Tulpule *et al*., 2006). In a previous study from our laboratory, using a full concentration of CHOP in mice with systemic NPM‐ALK^+^ T‐cell lymphoma resulted in significant toxic effects including therapy‐related death (George *et al*., 2014). In the current study, the effects of CHOP alone or in combination with TrkAi were analyzed, but we opted to use only half of the original concentration to avoid possible lethal toxicities. Of note is that CHOP appeared to induce superior effects on tumor size and mice survival than TrkAi alone. Nonetheless, the effects of low concentrations of CHOP or TrkAi alone were much more pronounced when the two therapies were combined. These results are not entirely unexpected considering that several previous studies concluded that the TrkA inhibitors could be more effective when combined with other therapies. For instance, the effects of CEP‐701 or chemotherapy were synergistically enhanced when the two regimens were combined to treat acute lymphoblastic leukemia, neuroblastoma, and breast cancer (Brown *et al*., 2006; Iyer *et al*., 2010; Zhang *et al*., 2015). In another study, enhancing SHP‐1 expression by using 5‐azacytidine improved the sensitivity of acute myeloid leukemia cells to CEP‐701 (Al‐Jamal *et al*., 2015). Moreover, combining the TrkA inhibitor CEP‐751 or CEP‐701 with androgen ablation was much more effective in treating prostate carcinoma *in vivo* than any of these regimens alone (George *et al*., 1999). It was also reported in a recent study that the TrkA inhibitors GW441756, K252a, CEP‐701, and Gö6976 promote the effects of agents that induce ROS‐mediated death in neuroblastoma cells (Ruggeri *et al*., 2014). The exact explanation for this phenomenon is not clear, but it is believed that using TrkA inhibitors alone may be effective only in tumors with activated Trk mutations (Thiele *et al*., 2009). However, it cannot be overlooked that developing potent and more selective TrkA inhibitors might significantly enhance their effects as monotherapeutic agents.

In conclusion, our data provide novel evidence that TrkA signaling contributes to NPM‐ALK^+^ T‐cell lymphoma pathogenesis. Selective and specific *in vitro* targeting of TrkA induced negative biological effects in these lymphoma cells. Using TrkAi as a monotherapy or combined with low concentrations of CHOP hindered the growth of the lymphoma tumors *in vivo* and improved mice survival. Although selective ALK inhibitors represent an emerging strategy to treat ALK^+^ neoplasms including NPM‐ALK^+^ T‐cell lymphoma (Mosse *et al*., 2013), several resistance mechanisms to these inhibitors have been already identified and characterized, which represents an important limitation (Dong *et al*., 2016; Isozaki *et al*., 2016; Katayama *et al*., 2016; Zdzalik *et al*., 2014). Our findings suggest that TrkA inhibition could represent an alternative therapeutic approach to tackle this aggressive neoplasm.

## Author contributions

WS, SKG, and BG designed and performed experiments, analyzed data, and contributed to writing the manuscript; AM performed experiments and analyzed data, CVC and SA provided essential experimental tools and analyzed data; HMA designed the investigation, performed research, analyzed data, supervised the study, and wrote the manuscript. All authors read and approved the manuscript as submitted.

## Supporting information


**Fig. S1.** The association and interactions between TrkA and NPM‐ALK.
