# Supplementary figures and images for: TrkA is a binding partner of NPM‐ALK that promotes the survival of ALK + T‐cell lymphoma
Source: Mol Oncol. 2017 Jun 18;11(9):1189–207. doi: 10.1002/1878-0261.12088 (PMC5579389; doi:10.1002/1878-0261.12088)

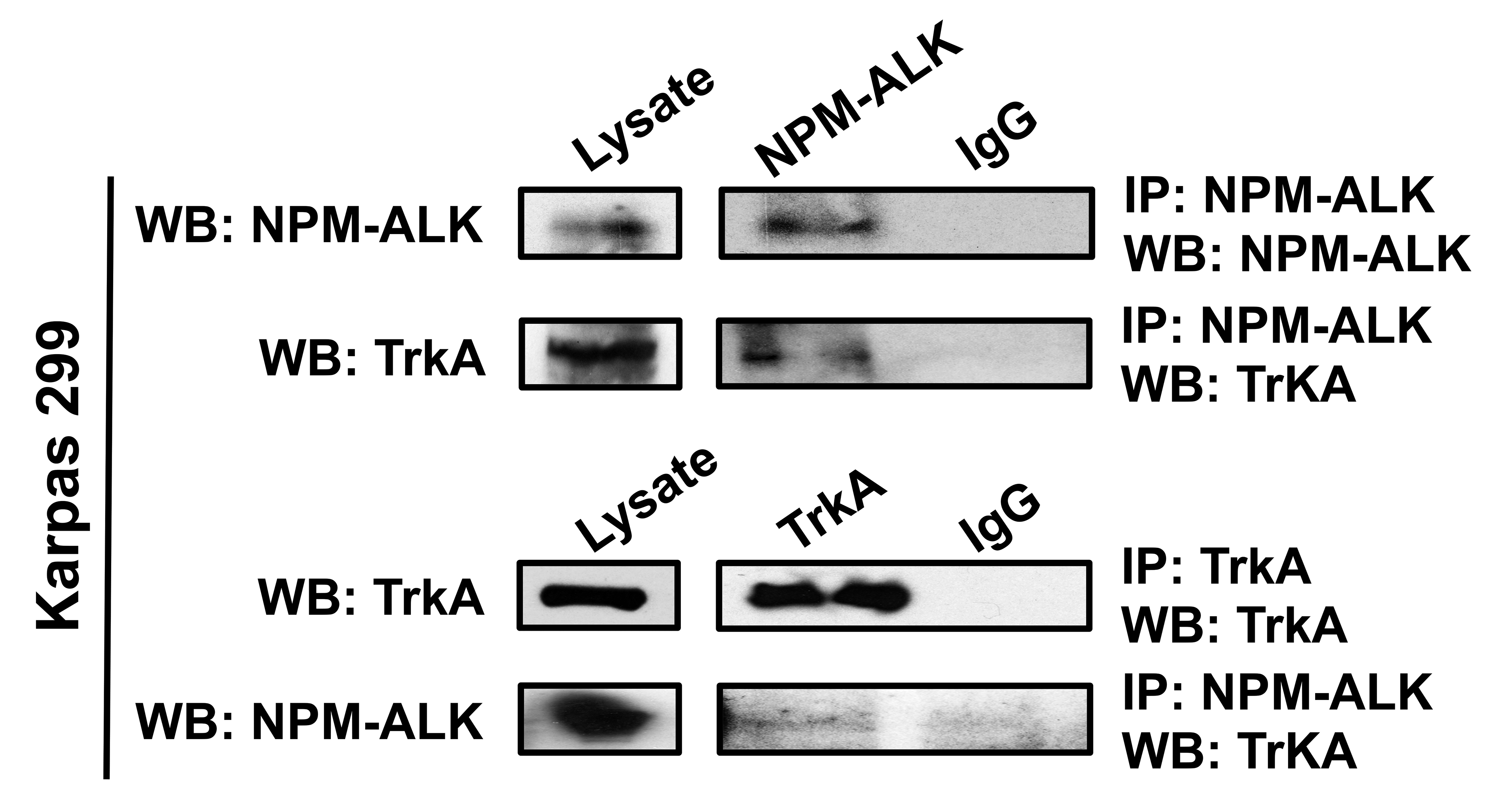


**1A**

**1C**


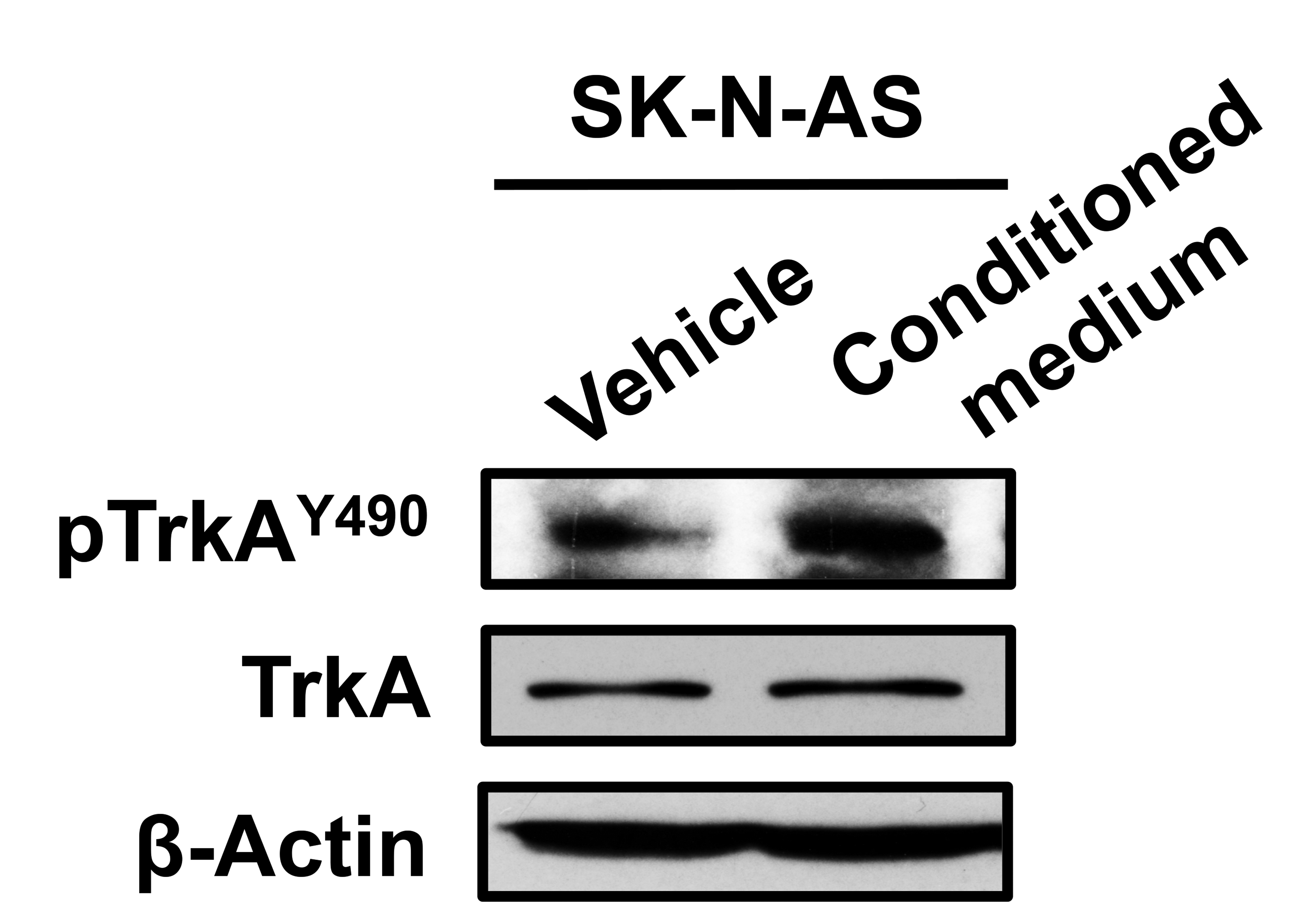

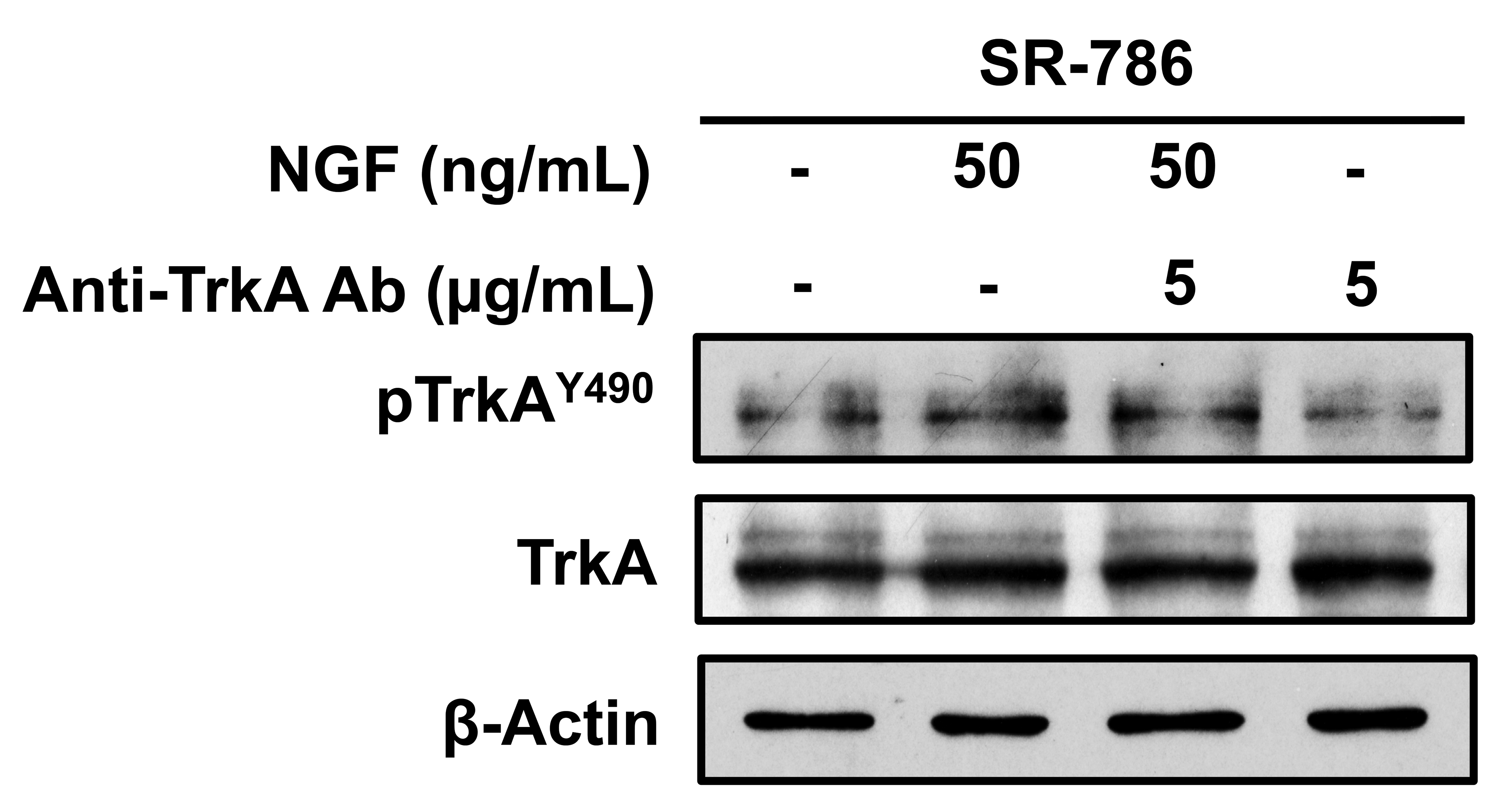


**1B**

Supplement: Supplementary file 1 — Fig. S1. The association and interactions between TrkA and NPM‐ALK. [file MOL2-11-1189-s001.docx]
